# Estimated Survival and Major Comorbidities of Very Preterm Infants Discharged Against Medical Advice vs Treated With Intensive Care in China

**DOI:** 10.1001/jamanetworkopen.2021.13197

**Published:** 2021-06-17

**Authors:** Siyuan Jiang, Xiangyuan Huang, Lan Zhang, Junyan Han, Yi Yang, Weiping Wang, Shoo K. Lee, Weili Yan, Yun Cao

**Affiliations:** 1 Department of Neonatology, Children’s Hospital of Fudan University, Shanghai, China; 2 Department of Clinical Epidemiology, Children’s Hospital of Fudan University, Shanghai, China; 3 National Health Commission, Key Laboratory of Neonatal Diseases (Fudan University), Children’s Hospital of Fudan University, Shanghai, China; 4 Maternal-Infant Care Research Centre and Department of Pediatrics, Mount Sinai Hospital, Toronto, Ontario, Canada; 5 Department of Pediatrics, University of Toronto, Toronto, Ontario, Canada; 6 Department of Obstetrics and Gynecology and Dalla Lana School of Public Health, University of Toronto, Toronto, Ontario, Canada

## Abstract

**Importance:**

A significant proportion of very preterm infants (<32 weeks’ gestation) are discharged against medical advice (DAMA) from neonatal intensive care units in China. There is minimal information available on the potential outcomes of providing complete care before discharge in these infants.

**Objective:**

To describe potential neonatal outcomes of DAMA in very preterm infants if they receive complete care based on estimates derived from a group of propensity score–matched infants who are not DAMA.

**Design, Setting, and Participants:**

This cohort study enrolled all infants born at between 24 and 31 weeks’ gestation from May 1, 2015, to April 30, 2018, and admitted to 25 tertiary neonatal intensive care units across China within 7 days of birth. A multilevel mixed-effects logistic regression model was constructed to estimate the propensity score for the likelihood of DAMA for each infant. Infants who were not DAMA were then matched to DAMA infants with the closest propensity score on a 1:1 ratio by using a nearest neighbor greedy matching algorithm without replacement. Incidences of neonatal outcomes were then calculated among the matched non-DAMA infants to simulate the outcomes of DAMA infants. Statistical analyses were performed from August 16, 2020, to September 26, 2020.

**Exposures:**

Discharge against medical advice, which was defined as termination of treatment and discharge before the treating physicians recommended discharge.

**Main Outcomes and Measures:**

Survival and survival without major morbidity.

**Results:**

The study enrolled a total of 14 083 infants (8141 boys [57.8%]) with a median gestational age of 30.1 weeks (interquartile range [IQR], 29.0-31.1 weeks) and a median birth weight of 1400 g (IQR, 1170-1600 g). Overall, 1876 of 14 083 very preterm infants (13.3%; 95% CI, 12.8%-13.9%) were DAMA, of whom 1367 of 1876 (72.9%; 95% CI, 70.8%-74.8%) required intensive care on discharge. A total of 1473 DAMA infants were successfully matched to 1473 non-DAMA infants. Overall, 1211 of 1473 matched non-DAMA infants (82.2%; 95% CI, 80.2%-84.1%) survived to discharge. The survival rates were 68.3% (95% CI, 62.4%-73.7%) for infants at 26 to 27 weeks’ gestation, 84.1% (95% CI, 80.7%-87.0%) for infants 28 to 29 weeks’ gestation, and 92.4% (95% CI, 90.0%-94.2%) for infants at 30 to 31 weeks’ gestation. A total of 872 of 1473 matched non-DAMA infants (59.2%; 95% CI, 56.7%-61.7%) survived without any major morbidity.

**Conclusions and Relevance:**

The results of this cohort study suggest that very preterm infants who are DAMA from neonatal intensive care units may have intact survival if complete care is provided. Efforts to reduce DAMA may be associated with improved outcomes of very preterm infants in China.

## Key Points

**Question:**

What are the estimated outcomes of very preterm infants in China treated with intensive care vs discharge against medical advice?

**Findings:**

In this cohort study of 14 083 very preterm infants in Chinese neonatal intensive care units, 13% of infants were discharged against medical advice. Overall, 82% of very preterm infants who were not discharged against medical advice and were propensity score–matched to infants who were discharged against medical advice survived, and 59% survived without any morbidity on discharge.

**Meaning:**

These findings suggest that very preterm infants in China who are discharged against medical advice may have intact survival if intensive care services are provided; efforts to reduce instances of discharge against medical advice may be associated with improved outcomes of very preterm infants in China.

## Teaser

This cohort study investigates neonatal outcomes of very preterm infants who are discharged against medical advice from neonatal intensive care units (NICUs) before complete care can be provided compared with infants who receive full intensive care treatment.

## Introduction

Discharge against medical advice (DAMA) refers to patients choosing to leave the hospital (or their caregivers choosing to have them leave) before the treating physicians recommend discharge.^1^ Discharge against medical advice exposes patients to the risk of inadequately treated medical problems and is associated with adverse outcomes.^1^ Several studies have reported high rates of DAMA among preterm infants, especially very preterm infants (<32 weeks’ gestation), in neonatal intensive care units (NICUs) in low- or middle-income countries,^2,3^ including China.^4,5,6,7,8,9,10,11^ For these most vulnerable and critically ill preterm infants, DAMA and incomplete care resulted in high mortality. Deaths after DAMA contributed to the mortality of preterm infants in China, accounting for 53% to 92% of deaths among preterm infants admitted to Chinese NICUs from 2007 to 2017.^4,5,6,7,8,9,10,11^ Therefore, reducing DAMA should be a priority to improve outcomes of preterm infants, especially very preterm infants, in China and other developing countries.

Many deaths among DAMA infants may be avoided and outcomes may be significantly improved if they receive complete care. However, to our knowledge, there has been no study investigating the potential outcomes of providing complete NICU care to DAMA infants. Our study attempted to match DAMA infants with non-DAMA infants using propensity score matching in a large contemporary cohort of very preterm infants in China, and we used the outcomes of matched non-DAMA infants to estimate the outcomes of DAMA infants if they received complete NICU care. We hypothesized that a considerable proportion of DAMA very preterm infants would survive if they received complete care.

## Methods

### Data Source

We used data from a large cohort of all preterm infants admitted to 25 tertiary NICUs in China from May 1, 2015, to April 30, 2018. Detailed clinical information of all preterm infants admitted to participating centers was prospectively collected using a standardized database. The database was initially established for a cluster randomized controlled trial, the Reduction of Infection in Neonatal Intensive Care Units Using the Evidence-Based Practice for Improving Quality (REIN-EPIQ) study.^10,12^ Twenty-five centers continuously collected data on all consecutively admitted preterm infants using this database for 3 years, from May 1, 2015, to April 30, 2018. All data collection followed standard operations and definitions and was performed by trained data abstractors.^10,12^ The study was approved by the Ethics Committee of the Children’s Hospital of Fudan University and recognized by all participating centers. A waiver of informed consent was approved owing to the use of deidentified patient data. The report of this study followed the Strengthening the Reporting of Observational Studies in Epidemiology (STROBE) reporting guideline.

### Sites

The 25 participating NICUs were from 19 provinces in China. All hospitals were public hospitals funded by government. Nineteen hospitals were teaching hospitals. Seventeen hospitals were national or provincial neonatal referral centers, and 8 were regional referral centers in metropolitan cities. The median number of level III NICU beds was 50 (range, 6-130 beds). All hospitals had the ability to provide comprehensive care for infants with gestational age younger than 28 weeks or birth weight less than 1000 g. During the study period, all provinces in China provided health care insurance for neonates. The cost of medical care in NICUs was partially covered by insurance with variable rates of subsidy in different provinces and areas, ranging from 50% to 80%.

### Study Population

All infants born at 24 to 31 weeks’ gestation between May 1, 2015, and April 30, 2018, and admitted to participating NICUs within 7 days of birth were included in the current study. Stillborn and delivery room deaths were not included. Infants with major congenital anomalies and infants transferred to nonparticipating hospitals with unknown outcomes were excluded. Infants were followed until death or discharge from the NICU. The outcome of DAMA infants after discharge was not followed owing to logistical difficulty.

### Definitions

Discharge against medical advice was defined as when parents or caregivers chose to terminate treatment and take the infants out of the hospital before the treating physicians recommended discharge. Infants who received complete care were classified as non-DAMA infants. Infants who were moribund on discharge and were taken out of hospital for palliative care were classified as in-hospital death. If DAMA infants required invasive or noninvasive mechanical ventilation, inotrope infusion, or total parenteral nutrition (no enteral feeds initiated) on the day of discharge, they were considered to require intensive care on discharge.

Gestational age was determined using the hierarchy of best obstetric estimate based on prenatal ultrasonography, menstrual history, obstetric examination, or all 3 methods of evaluation. If the obstetric estimate was not available or was different from the postnatal pediatric estimate of gestation by more than 2 weeks, the gestational age was estimated using the Ballard Score.^13^ Small for gestational age was defined as birth weight less than the 10th percentile for gestational age according to the Chinese neonatal birth weight values.^14^ Prenatal care was defined as at least 1 pregnancy-related hospital visit during pregnancy. Antenatal corticosteroid treatment was defined as a partial or complete course of antenatal corticosteroids before birth. The Transport Risk Index of Physiologic Stability (TRIPS) score^15,16^ was used as an illness severity score on NICU admission. The TRIPS score included an assessment of temperature, respiratory status, systolic blood pressure, response to noxious stimuli on admission, and was shown to be associated with 7-day and overall NICU mortality.^15,16^

### Outcomes

Survival without morbidity was defined as survival without any of 4 major morbidities, including necrotizing enterocolitis, intraventricular hemorrhage or periventricular leukomalacia, retinopathy of prematurity, and bronchopulmonary dysplasia. Necrotizing enterocolitis was defined as greater than or equal to stage 2 according to the Bell criteria.^17,18^ Intraventricular hemorrhage was defined as greater than or equal to grade 3 according to the Papile criteria.^19^ Periventricular leukomalacia was defined as the presence of periventricular cysts on cranial ultrasonography or cranial magnetic resonance imaging (MRI) scans. Bronchopulmonary dysplasia was defined as mechanical ventilation or oxygen dependency at 36 weeks’ postmenstrual age.^20^ Retinopathy of prematurity was defined as greater than or equal to stage 3 according to the International Classification of Retinopathy of Prematurity.^21^

### Statistical Analysis

Differences of baseline characteristics between DAMA and non-DAMA groups were assessed using Pearson χ^2^ test for categorical data and the Wilcoxon rank sum test for continuous data. It was impossible to directly summarize the outcomes of DAMA infants, because medical care was terminated prematurely for these infants. Therefore, we proposed to identify a group of non-DAMA infants with similar baseline characteristics as DAMA infants, and the outcomes of this group of non-DAMA infants were used to simulate the outcomes of DAMA infants per the hypothesis that DAMA infants received complete medical care in NICUs. Because DAMA was not a randomly assigned exposure and significant differences in baseline characteristics existed between DAMA and non-DAMA infants, we used propensity score matching to identify non-DAMA infants with comparable baseline characteristics to those of DAMA infants. A multilevel mixed-effects logistic regression model was constructed to estimate the propensity score for the likelihood that each infant would be DAMA, accounting for the correlations among the infants within sites. Infant-level candidate variables included gestational age, birth weight, sex, small for gestational age, primigravida, Apgar score less than 3 at 5 minutes, illness severity score (TRIPS score on admission), cesarean delivery, maternal hypertension, maternal diabetes, antenatal steroids, and delivery in tertiary perinatal centers. Variables associated with DAMA via univariable analysis with a threshold of *P* < .20 were included in the final multilevel mixed-effects logistic model.

Non-DAMA infants were then matched to DAMA infants with the closest propensity score on a 1:1 ratio by using the nearest neighbor greedy matching algorithm without replacement. The maximum distance between 2 matched infants (the caliper) for the study was set to 0.25. We evaluated baseline characteristic imbalances between the matched DAMA and non-DAMA infants by standardized differences. We defined the standardized difference for a continuous variable as the absolute difference in group means divided by an estimate of the pooled SD. The derivation was similar for nominal variables. We considered a standardized difference less than 0.10 as negligible imbalance between groups. Rates of neonatal outcomes were then calculated among the matched non-DAMA infants to simulate the outcomes of DAMA infants.

The majority of DAMA infants were discharged within 7 days after birth, during which period perinatal characteristics might have the strongest association with DAMA decision. Therefore, we repeated the same analysis for the subgroup of DAMA infants who were discharged within 7 days after birth.

All analyses were performed from August 16, 2020, to September 26, 2020, using Stata/SE, version 15.0 (StataCorp) with the psmatch2 package. All *P* values were 2-sided, and *P* < .05 was considered significant.

## Results

### Incidence of DAMA

A total of 14 083 infants (8141 boys [57.8%]), with a median gestational age of 30.1 weeks (interquartile range [IQR], 29-31.1 weeks) and a median birth weight of 1400 g (IQR, 1170-1600 g) were enrolled in the study after excluding 571 infants from all 14 654 very premature infants admitted to participating NICUs within 7 days after birth during the study period (Figure).

**Figure.**
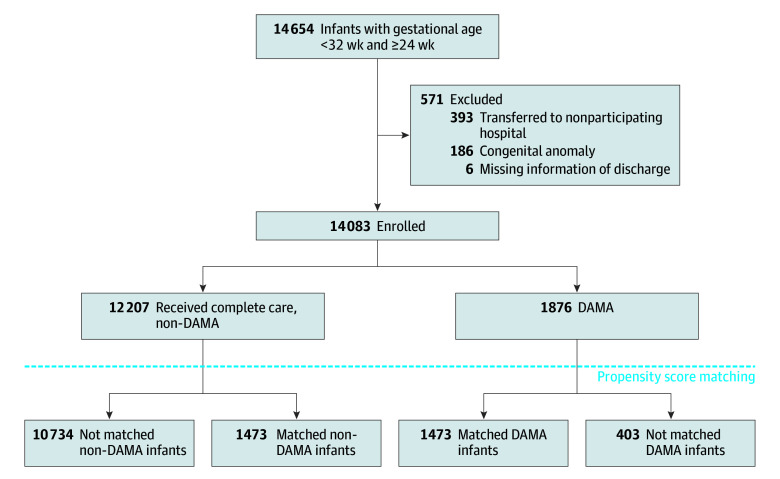
Study Population DAMA indicates discharged against medical advice.

Overall, 12 207 of 14 083 infants (86.7%; 95% CI, 86.1%-87.2%) received complete care (non-DAMA), and 1876 of 14 083 infants (13.3%; 95% CI, 12.8%-13.9%) were DAMA. The DAMA rate generally increased with decreasing gestational age. The DAMA rates by gestational age were as follows: 15 of 881 infants (18.5%; 95% CI, 11.4%-28.5%) born at 24 weeks’ gestation, 55 of 198 infants (27.8%; 95% CI, 22.0%-34.4%) born at 25 weeks’ gestation, 134 of 502 infants (26.7%; 95% CI, 23.0%-30.7%) born at 26 weeks’ gestation, 206 of 915 infants (22.5%; 95% CI, 19.9%-25.3%) born at 27 weeks’ gestation, 296 of 1797 infants (16.5%; 95% CI, 14.8%-18.3%) born at 28 weeks’ gestation, 387 of 2569 infants (15.1%; 95% CI, 13.7%-16.5%) born at 29 weeks’ gestation, 365 of 3403 infants (10.7%; 95% CI, 9.7%-11.8%) born at 30 weeks’ gestation, and 418 of 4618 infants (9.1%; 95% CI, 8.3%-9.9%) born at 31 weeks’ gestation (eFigure 1 in Supplement 1).

Overall, 1367 of 1876 DAMA infants (72.9%; 95% CI, 70.8%-74.8%) required intensive care on discharge (Table 1). The proportions of DAMA infants requiring intensive care increased with decreasing gestational age (Table 1). As many as 617 of 706 infants (87.4%; 95% CI, 84.7%-89.7%) younger than 29 weeks’ gestation were on intensive care at discharge, and 477 of 783 infants (60.9%; 95% CI, 57.4%-64.3%) at 30 to 31 weeks gestation also required intensive care at the time of discharge.

**Table 1.** Treatment on Discharge for Infants Discharged Against Medical Advice

Variable	No. (%)								
	Total (N = 1876)	Gestational age, wk							
		24 (n = 15)	25 (n = 55)	26 (n = 134)	27 (n = 206)	28 (n = 296)	29 (n = 387)	30 (n = 365)	31 (n = 418)
Intensive care^a^	1367 (72.9)	15 (100)	51 (92.7)	122 (91.0)	179 (86.9)	250 (84.5)	273 (70.5)	230 (63.0)	247 (59.1)
Invasive ventilation	697 (37.2)	12 (80.0)	31 (56.4)	75 (56.0)	92 (44.7)	143 (48.3)	130 (33.6)	107 (29.3)	107 (25.6)
Noninvasive ventilation	602 (32.1)	3 (20.0)	18 (32.7)	48 (35.8)	76 (36.9)	105 (35.5)	124 (32.0)	112 (30.7)	116 (27.8)
Inotropes	326 (17.4)	2 (13.3)	17 (30.9)	31 (23.1)	45 (21.8)	55 (18.6)	74 (19.1)	57 (15.6)	45 (10.8)
Total parenteral nutrition	636 (33.9)	9 (60.0)	24 (43.6)	51 (38.1)	86 (41.7)	123 (41.6)	128 (33.1)	103 (28.2)	112 (26.8)

^a^ Intensive care on discharge refers to use of invasive ventilation, noninvasive ventilation, inotropes or total parenteral nutrition on discharge.

### Characteristics Associated With DAMA

Baseline characteristics of non-DAMA infants and DAMA infants are listed in Table 2. Overall, DAMA infants were of younger gestational age (median, 29.4 weeks [IQR, 28.0-30.7 weeks] vs 30.3 weeks [IQR, 29.0-31.1 weeks]), of lesser birth weight (median, 1250 g [IQR, 1040-1500 g] vs 1400 g [IQR, 1200-1620 g]), were less likely to be boys (992 of 1876 [52.9%] vs 7149 of 12 207 [58.6%]), and were more likely to be small for gestational age (230 of 1876 [12.3%] vs 1263 of 12 207 [10.3%]) compared with infants who received complete care. In addition, DAMA infants were more likely to have a low Apgar score after birth (50 of 1594 [2.9%] vs 168 of 11 068 [1.4%]) and a higher TRIPS score on admission (median, 19 points [IQR, 12-28 points] vs 13 points [IQR, 7-21 points]). Mothers of DAMA infants were less likely to deliver via cesarean delivery (638 of 1875 [34.0%] vs 5722 of 12 204 [46.9%]), to receive prenatal care (1794 of 1855 [96.7%] vs 11 945 of 12 107 [98.7%]) and antenatal steroids (969 of 1781 [54.4%] vs 8107 of 11 627 [69.7%]), and to have diabetes (140 of 183 [7.6%] vs 1458 of 12 075 [12.1%]).

**Table 2.** Infant and Maternal Characteristics of DAMA Infants and Non-DAMA Infants

Variable	Before PS match				After PS match				
	No. (%)		*P* value	Standardized difference	No. (%)		*P* value	Standardized difference
	Non-DAMA (n = 12207)	DAMA (n = 1876)			Non-DAMA (n = 1473)	DAMA (n = 1473)		
Gestational age, median (IQR), wk	30.3 (29.0-31.1)	29.4 (28.0-30.7)	<.001	0.42	29.4 (28.0-30.7)	29.4 (28.0-30.7)	.87	0.02
<28	1286 (10.5)	410 (21.9)			327 (22.2)	320 (21.7)		
28-29	3683 (30.2)	683 (36.4)			516 (35.0)	540 (36.7)		
30-31	7238 (59.3)	783 (41.7)			630 (42.8)	613 (41.6)		
Birth weight, median (IQR), g	1400 (1200-1620)	1250 (1040-1500)	<.001	0.44	1250 (1050-1480)	1250 (1030-1500)	.59	0.00
<1000	1094 (9.0)	356 (19.0)			258 (17.5)	296 (20.1)		
1000-1499	6263 (51.3)	1035 (55.2)			860 (58.4)	800 (54.3)		
≥1500	4849 (39.7)	485 (25.8)			355 (24.1)	377 (25.6)		
Boys	7149 (58.6)	992 (52.9)	<.001	0.12	803 (54.5)	773 (52.5)	.27	0.04
Girls	5058 (41.4)	884 (47.1)	<.01	0.12	670 (45.5)	700 (47.5)	.27	0.04
SGA	1263 (10.3)	230 (12.3)	.012	0.07	183 (12.4)	187 (12.7)	.82	0.01
Primigravida	4316/12193 (35.4)	643/1876 (34.3)	.34	0.05	487 (33.1)	494 (33.5)	.79	0.01
Inborn	8352 (68.4)	1241 (66.2)	.05	0.00	1087 (73.8)	1096 (74.4)	.71	0.01
5-min Apgar score ≤3	168/11068 (1.4)	50/1594 (2.9)	<.001	0.10	38 (2.6)	41 (2.8)	.73	0.01
TRIPS score, median (IQR)	13 (7-21)	19 (12-28)	<.001	0.42	19 (12-28)	19.0 (12-28)	.44	0.03
Cesarean delivery	5722/12204 (46.9)	638/1875 (34.0)	<.001	0.26	570 (38.7)	543 (36.9)	.3	0.04
Prenatal care	11945/12107 (98.7)	1794/1855 (96.7)	<.001	0.07	1453 (98.6)	1447 (98.2)	.37	0.03
Antenatal steroids	8107/11627 (69.7)	969/1781 (54.4)	<.001	0.32	873 (59.3)	852 (57.8)	.43	0.03
Maternal hypertension	1729/12089 (14.3)	237/1836 (12.9)	.11	0.04	211 (14.3)	198 (13.4)	.49	0.02
Maternal diabetes	1458/12075 (12.1)	140/1835 (7.6)	<.001	0.16	130 (8.8)	117 (7.9)	.39	0.03

Abbreviations: DAMA, discharge against medical advice; IQR, interquartile range; PS, propensity score; SGA, small for gestational age; TRIPS, Transport Risk Index of Physiologic Stability score.

The propensity modeling of DAMA was shown in eTable 1 in Supplement 1. A total of 1473 of 1876 DAMA infants (78.5%) were successfully matched to 1473 non-DAMA infants by propensity score matching (Figure). Baseline characteristics achieved adequate balance between matched DAMA and non-DAMA infants after matching (Table 2). A total of 403 of 1876 DAMA infants (21.5%) failed to match to non-DAMA infants. The comparison of characteristics between matched and nonmatched DAMA infants as well as matched and nonmatched non-DAMA infants are shown in eTable 2 in Supplement 1.

### Outcomes of Matched Non-DAMA Infants

Overall, 1211 of 1473 matched non-DAMA infants (82.2%; 95% CI, 80.2%-84.1%) survived to discharge (Table 3). The survival rates were 68.3% (95% CI, 62.4%-73.7%) for infants at 26 to 27 weeks’ gestation, 84.1% (95% CI, 80.7%-87.0%) for infants at 28 to 29 weeks’ gestation, and 92.4% (95% CI, 90.0%-94.2%) for infants at 30 to 31 weeks’ gestation. A total of 872 of 1473 matched non-DAMA infants (59.2%; 95% CI, 56.7%-61.7%) survived without morbidity in the NICU. The rates of survival without morbidity also increased with increasing gestational age, and 306 of 516 infants born at 28 to 29 weeks’ gestation (59.3%; 95% CI, 55.0%-63.5%) and 457 of 630 infants born at 30 to 31 weeks’ gestation (72.5%; 95% CI, 68.9%-75.9%) had intact survival on discharge. Bronchopulmonary dysplasia (235 of 1211 [19.4%]) and severe intraventricular hemorrhage or periventricular leukomalacia (141 of 1315 [10.7%]) were the most common morbidities. The incidences of neonatal outcomes among matched non-DAMA infants compared with all non-DAMA infants are shown in eFigure 2 in Supplement 1.

**Table 3.** In-Hospital Outcomes and NICU Treatments of Matched Non-DAMA Infants

Variable	No. (%)								
	Total (N = 1473)	Gestational age, wk							
		24 (n = 26)	25 (n = 42)	26 (n = 104)	27 (n = 155)	28 (n = 246)	29 (n = 270)	30 (n = 304)	31 (n = 326)
Survival without morbidity^a^	872 (59.2)	1 (3.8)	6 (14.3)	33 (31.7)	69 (44.5)	133 (54.1)	173 (64.1)	212 (69.7)	245 (75.2)
Survival rate	1211 (82.2)	4 (15.4)	14 (33.3)	66 (63.5)	111 (71.6)	201 (81.7)	233 (86.3)	280 (92.1)	302 (92.6)
IVH grade III or above or PVL^b^	141/1315 (10.7)	6/15 (40.0)	8/29 (27.6)	20/88 (22.7)	20/132 (15.2)	35/224 (15.6)	19/243 (7.8)	19/289 (6.7)	14/299 (4.7)
NEC stage II or above	87 (5.9)	3 (11.5)	3 (7.1)	5 (4.8)	8 (5.2)	15 (6.1)	19 (7.0)	18 (5.9)	16 (4.9)
BPD^c^	235/1211 (19.4)	3/4 (75.0)	7/14 (50.0)	21/66 (31.8)	30/111 (27.0)	39/201 (19.4)	45/233 (19.3)	49/280 (17.5)	41/302 (13.6)
ROP stage III or greater^d^	30/1031 (2.8)	3/6 (50.0)	3/15 (20.0)	7/66 (10.6)	8/108 (7.4)	3/190 (1.6)	2/213 (0.9)	3/237 (1.3)	1/226 (0.4)

Abbreviations: BPD, bronchopulmonary dysplasia; DAMA, discharge against medical advice; IVH, intraventricular hemorrhage; NEC, necrotizing enterocolitis; NICU, neonatal intensive care units; PVL, periventricular leukomalacia; ROP, retinopathy of prematurity.

^a^ Morbidities include NEC stage II or above, IVH grade III or above or PVL, BPD and ROP stage III or above.

^b^ Rate of IVH grade III or above or PVL was calculated among infants with neuroimaging results.

^c^ BPD was defined as mechanical ventilation or oxygen dependency at 36 weeks’ postmenstrual age. Rate of BPD was calculated among infants with known respiratory support status at 36 weeks’ postmenstrual age.

^d^ Rate of ROP was calculated among infants with eye examination in NICU.

### Infants DAMA Within 7 Days After Birth

A total of 1031 of 1876 DAMA infants (55.0%; 95% CI, 52.7%-57.2%) were discharged within 7 days after birth. At discharge, 956 of 1031 infants (92.7%; 95% CI, 91.0%-94.2%) were still in intensive care (eTable 3 in Supplement 1). The results of propensity score matching and outcomes of matched non-DAMA infants to infants DAMA within 7 days were similar to those of all DAMA infants (eTables 4-6 in Supplement 1).

## Discussion

To our knowledge, this is the first study examining the potential outcomes of DAMA infants if they received adequate care. We found that DAMA occurred in 13% of very preterm infants admitted to Chinese NICUs, among whom 73% were still on intensive medical support at discharge. However, 82% of non-DAMA very preterm infants who were matched with DAMA infants with similar baseline characteristics survived, and 59% survived without any morbidity on NICU discharge. Such results convey important information about the potential improvement in clinical outcomes for DAMA infants should they receive complete care and suggests the need for efforts to reduce DAMA.

We found a 13% rate of DAMA among very preterm infants in our study, which is lower than the 15% to 20% incidence rate reported by previous studies from China.^5,10^ However, the 13% DAMA rate remained significant and suggests that understanding this phenomenon is an important issue in perinatal health care. A few previous reports investigated reasons for DAMA in Chinese pediatric intensive care units or NICUs.^5,6,22,23^ The main reported reasons included inability to afford medical costs and caregiver concern about poor prognosis. However, the retrospective designs of these studies may have compromised their reliability, and their results were conflicting. Also, many dynamic factors, such as the changing health insurance policies with increasing subsidies for neonatal intensive care, the revocation of population control measures in China, and the rapid improvement of neonatal care, may influence the decision-making process involved in DAMA. A well-designed, prospective survey is needed to investigate concurrent modifiable factors affecting DAMA rates and to guide the efforts to reduce DAMA rates.

We did not follow up infants after DAMA to determine actual mortality after discharge. However, we found that approximately 73% of DAMA infants remained in intensive care including invasive or noninvasive ventilation, inotropic therapy, and total parenteral nutrition at the time of discharge. These infants were highly likely to die after discharge. Additionally, suboptimal temperature control, inappropriate feeding practices, infection, and other conditions would also compromise the chances for survival of these vulnerable infants after DAMA. Therefore, the mortality rate of DAMA infants after discharge may be even higher. In our cohort, a total of 960 very preterm infants died in the hospital, whereas at least 1367 DAMA infants on intensive care at discharge may die after discharge. Discharge against medical advice may account for up to 60% of very preterm infant deaths, which is similar to the findings of previous studies.^5,6,7,9^ Therefore, our study results suggest that DAMA infants should be a high-priority target population for interventions to reduce the overall mortality of very preterm infants in China.

We found that DAMA infants were smaller and sicker on admission compared with non-DAMA infants. The delivery of DAMA infants was also more likely to occur in an uncontrolled environment, eg, the infants were less likely to receive antenatal steroids. These baseline perinatal characteristics of DAMA infants could increase the risks of adverse outcomes. Therefore, using propensity score matching to identify a group of non-DAMA infants with similar baseline characteristics to DAMA infants and whose outcomes are known may mitigate this problem.

Assuming that DAMA infants would have a survival rate similar to matched infants (82%) if they received complete care, and assuming that the 73% of DAMA infants who required intensive care at discharge would die, then mortality among DAMA infants would decrease by 75% if DAMA were eliminated. Because DAMA infants accounted for 13% of all admitted infants, this would translate to a 7% absolute reduction of overall mortality for all very preterm infant admissions.

### Limitations

There were several limitations in our study. First, there may have been unmeasured factors associated with DAMA status and neonatal outcomes. However, we included all well-known perinatal characteristics proven to be associated with neonatal outcomes. Other factors, such as parental socioeconomic status, were likely to have more influence on long-term instead of short-term outcomes of preterm infants. Second, instances when DAMA infants were not matched to non-DAMA infants may have resulted in selection bias. Compared with matched DAMA infants, the nonmatched DAMA infants were less likely to be delivered in tertiary perinatal centers, be delivered by cesarean delivery, and to receive prenatal care and antenatal steroids, and they had a lower Apgar score. Therefore, the estimated survival of matched DAMA infants may have overestimated the survival of the entire DAMA population. Third, the actual outcomes of DAMA infants after discharge were not monitored, and our mortality estimates based on treatment at discharge may be an underestimate. Fourth, we did not investigate the reasons for DAMA among affected families.

## Conclusions

Our study results suggest that very preterm infants who are DAMA from Chinese NICUs may have intact survival if complete care is provided. Results further suggest that efforts aimed at reducing DAMA rates are essential and urgent and may be associated with an improvement in the overall outcomes of very preterm infants in China.

## Notes

Supplement 1. **eTable 1.** Propensity Modeling of Discharge Against Medical Advice **eTable 2.** Infant and Maternal Characteristics of Matched and Nonmatched DAMA Infants After PS Matching **eTable 3.** Treatment on Discharge for Infants Discharged Against Medical Advice Within 7 Days After Birth **eTable 4.** Infant and Maternal Characteristics of Infants DAMA in 7 Days and Non-DAMA Infants **eTable 5.** Propensity Modeling of Discharge Against Medical Advice Within 7 Days After Birth **eTable 6.** In-Hospital Outcomes and NICU Treatments of Matched Non-DAMA Infants for Infants DAMA in 7 Days **eFigure 1.** Rates of Discharge Against Medical Advice by Gestational Age **eFigure 2.** Incidences of Neonatal Outcomes With 95% Confidence Intervals Among Matched Non-DAMA Infants and all Non-DAMA Infants

Supplement 2.
Reduction of Infection in Neonatal Intensive Care Units Using the Evidence-Based Practice for Improving Quality (REIN-EPIQ) Study Group Members and Site Investigators
