# Supplementary material for: Estimated Survival and Major Comorbidities of Very Preterm Infants Discharged Against Medical Advice vs Treated With Intensive Care in China
Source: JAMA Netw Open. 2021 Jun 17;4(6):e2113197. doi: 10.1001/jamanetworkopen.2021.13197 (PMC12543406; doi:10.1001/jamanetworkopen.2021.13197)
Supplement: Supplement 1. — eTable 1. Propensity Modeling of Discharge Against Medical Advice eTable 2. Infant and Maternal Characteristics of Matched and Nonmatched DAMA Infants After PS Matching eTable 3. Treatment on Discharge for Infants Discharged Against Medical Advice Within 7 Days After Birth eTable 4. Infant and Maternal Characteristics of Infants DAMA in 7 Days and Non-DAMA Infants eTable 5. Propensity Modeling of Discharge Against Medical Advice Within 7 Days After Birth eTable 6. In-Hospital Outcomes and NICU Treatments of Matched Non-DAMA Infants for Infants DAMA in 7 Days eFigure 1. Rates of Discharge Against Medical Advice by Gestational Age eFigure 2. Incidences of Neonatal Outcomes With 95% Confidence Intervals Among Matched Non-DAMA Infants and all Non-DAMA Infants [file jamanetwopen-e2113197-s001.pdf]

Supplementary Online Content

Jiang S, Huang X, Zhang L, et al; Reduction of Infection in Neonatal Intensive Care Units Using the Evidence-Based Practice for Improving Quality (REIN-EPIQ) Study Group. Estimated survival and major comorbidities of very preterm infants discharged against medical advice vs treated with intensive care in China. *JAMA Netw Open*. 2021;4(6):e2113197. doi:10.1001/jamanetworkopen.2021.13197

eTable 1. Propensity Modeling of Discharge Against Medical Advice
eTable 2. Infant and Maternal Characteristics of Matched and Nonmatched DAMA Infants After PS Matching
eTable 3. Treatment on Discharge for Infants Discharged Against Medical Advice Within 7 Days After Birth
eTable 4. Infant and Maternal Characteristics of Infants DAMA in 7 Days and Non-DAMA Infants
eTable 5. Propensity Modeling of Discharge Against Medical Advice Within 7 Days After Birth
eTable 6. In-Hospital Outcomes and NICU Treatments of Matched Non-DAMA Infants for Infants DAMA in 7 Days
eFigure 1. Rates of Discharge Against Medical Advice by Gestational Age
eFigure 2. Incidences of Neonatal Outcomes With 95% Confidence Intervals Among Matched Non-DAMA Infants and all Non-DAMA Infants

This supplementary material has been provided by the authors to give readers additional information about their work.

© 2021 Jiang S et al. *JAMA Network Open.*

eTable 1. Propensity Modeling of Discharge Against Medical Advice

Variable	Univariable Analysis			Multivariable Analysis[*]	
	OR (95%CI)	P-value		aOR (95%CI)	P-value
Gestational age	0.79 (0.77-0.81)	<0.001		0.86 (0.81-0.91)	<0.001
Birth weight[#]	0.87 (0.86-0.89)	<0.001		0.90 (0.87-0.93)	<0.001
Male	0.80 (0.72-0.88)	<0.001		0.76 (0.67-0.86)	<0.001
SGA	1.21 (1.04-1.40)	<0.001		1.07 (0.86-1.33)	0.544
Primigravida	0.95 (0.86-1.05)	0.344		-	-
Inborn	0.90 (0.81-1.00)	0.05		0.83 (0.68-1.0)	0.059
5-min Apgar score ≤3	2.10 (1.53-2.90)	<0.001		1.32 (0.90-1.93)	0.155
TRIPS score	1.03 (1.03-1.04)	<0.001		1.01 (1.00-1.02)	<0.001
Cesarean section	0.58 (0.53-0.65)	<0.001		0.88 (0.77-1.02)	0.08
Prenatal care	0.40 (0.30-0.54)	<0.001		0.97 (0.50-1.29)	0.354
Antenatal steroids	0.52 (0.47-0.57)	<0.001		0.67 (0.59-0.76)	<0.001
Maternal hypertension	0.89 (0.77-1.03)	0.11		0.97 (0.79-1.17)	0.694
Maternal diabetes	0.60 (0.50-0.72)	<0.001		0.76 (0.62-0.94)	<0.001

*Multilevel mixed-effects logistic regression model accounting for the correlations among the infants within sites

[#]Every 100g increase

Abbreviations: OR, odds ratio; aOR adjusted odds ratio; 95%CI, 95% confidence interval; SGA, small for gestational age; TRIPS score, Transport Risk Index of Physiologic Stability score

© 2021 Jiang S et al. *JAMA Network Open.*

eTable 2. Infant and Maternal Characteristics of Matched and Nonmatched DAMA Infants After PS Matching

	Non-matched DAMA infants	Matched DAMA infants	p-value
	N=403	N=1473	
Gestational age, median (IQR)	29.4 (28.0, 30.7)	29.4 (28.0, 30.7)	0.71
Birth weight, median (IQR)	1300 (1100, 1500)	1250 (1030, 1500)	0.045
Male, n (%)	219 (54.3%)	773 (52.5%)	0.51
SGA, n (%)	43 (10.7%)	187 (12.7%)	0.27
Primigravida, n (%)	149 (37.0%)	494 (33.5%)	0.20
Inborn, n (%)	145 (36.0%)	1096 (74.4%)	<0.001
5-min Apgar score ≤3, n (%)	9/121(7.4%)	41/1473 (2.8%)	0.005
TRIPS score, median (IQR)	19 (12, 28)	19 (12, 28)	0.87
Cesarean section, n (%)	95/402 (23.6%)	543/1473 (36.9%)	<0.001
Prenatal care, n (%)	347/382(90.8%)	1447/1473 (98.2%)	<0.001
Antenatal steroids, n (%)	117/308 (38.0%)	852/1473 (57.8%)	<0.001
Maternal hypertension, n (%)	39/363 (10.7%)	198/1473 (13.4%)	0.17
Maternal diabetes, n (%)	23/362 (6.4%)	117/1473 (7.9%)	0.31

Abbreviations: PS, propensity score; DAMA, discharge against medical advice; IQR, interquartile range; SGA, small for gestational age; TRIPS score, Transport Risk Index of Physiologic Stability score

© 2021 Jiang S et al. *JAMA Network Open.*

eTable 3. Treatment on Discharge for Infants Discharged Against Medical Advice Within 7 Days After Birth

	24 weeks	25 weeks	26 weeks	27 weeks	28 weeks	29 weeks	30 weeks	31 weeks	Total
	N=10	N=36	N=80	N=125	N=177	N=211	N=174	N=218	N=1031
Invasive Ventilation, n (%)	8 (80.0%)	25 (69.4%)	50 (62.5%)	61 (48.8%)	99 (55.9%)	92 (43.6%)	80 (46.0%)	76 (34.9%)	491 (47.6%)
Non-invasive Ventilation, n (%)	2 (20.0%)	10 (27.8%)	28 (35.0%)	48 (38.4%)	73 (41.2%)	91 (43.1%)	66 (37.9%)	91 (41.7%)	409 (39.7%)
Inotropes, n (%)	1 (10.0%)	16 (44.4%)	25 (31.2%)	31 (24.8%)	40 (22.6%)	52 (24.6%)	38 (21.8%)	35 (16.1%)	238 (23.1%)
TPN, n (%)	9 (90.0%)	23 (63.9%)	50 (62.5%)	82 (65.6%)	113 (63.8%)	120 (56.9%)	93 (53.4%)	101 (46.3%)	591 (57.3%)
Intensive care, n (%)	10 (100.0%)	36 (100.0%)	78 (97.5%)	122 (97.6%)	170 (96.0%)	201 (95.3%)	155 (89.1%)	184 (84.4%)	956 (92.7%)

Abbreviations: DAMA, discharge against medical advice; TPN, total parenteral nutrition

© 2021 Jiang S et al. *JAMA Network Open.*

eTable 4. Infant and Maternal Characteristics of Infants DAMA in 7 Days and Non-DAMA Infants

	Before PS match					After PS match			
	Non-DAMA	DAMA	p-value	Standardized Difference		Non-DAMA	DAMA	p-value	Standardized Difference
	N=12207	N=1031				N=801	N=801		
Gestational age, median (IQR)	30.3 (29.0, 31.1)	29.3 (28.0, 30.6)	<0.001	0.49		29.4 (28.0, 30.6)	29.3 (28.0, 30.6)	0.63	0.003
Birth weight, median (IQR)	1400 (1200, 1620)	1200 (1000, 1450)	<0.001	0.52		1230 (1050, 1450)	1200 (1000, 1450)	0.30	0.02
Male, n (%)	7149 (58.6%)	521 (50.5%)	<0.001	0.16		404 (50.4%)	402 (50.2%)	0.92	0.005
SGA, n (%)	1263 (10.3%)	137 (13.3%)	0.003	0.11		108 (13.5%)	111 (13.9%)	0.83	0.01
Primigravida, n (%)	4316/12193 (35.4%)	342 (33.2%)	0.15	0.06		274 (34.2%)	263 (32.8%)	0.56	0.03
Inborn, n (%)	8352 (68.4%)	699 (67.8%)	0.68	0.03		594 (74.2%)	605 (75.5%)	0.53	0.03
5-min Apgar score ≤3, n (%)	168/11068 (1.4%)	39 (4.5%)	<0.001	0.16		26 (3.2%)	32 (4.0%)	0.42	0.05
TRIPS score, median (IQR)	13 (7, 21)	20 (12, 28)	<0.001	0.52		19 (12, 28)	21 (12, 28)	0.77	0.005
Cesarean section, n (%)	5722/12204 (46.9%)	313 (30.4%)	<0.001	0.36		266 (33.2%)	259 (32.3%)	0.71	0.02
Prenatal care, n (%)	11945/12107 (98.7%)	986 (96.6%)	<0.001	0.11		784 (97.9%)	783 (97.8%)	0.86	0.01
Antenatal steroids, n (%)	8107/11627 (69.7%)	485 (49.6%)	<0.001	0.45		427 (53.3%)	415 (51.8%)	0.55	0.03
Maternal hypertension, n (%)	1729/12089 (14.3%)	135 (13.4%)	0.41	0.02		120 (15.0%)	114 (14.2%)	0.67	0.02
Maternal diabetes, n (%)	1458/12075 (12.1%)	70 (6.9%)	<0.001	0.21		50 (6.2%)	53 (6.6%)	0.76	0.01

Abbreviations: PS, propensity score; DAMA, discharge against medical advice; IQR, interquartile range; SGA, small for gestational age; TRIPS score, Transport Risk Index of Physiologic Stability score

© 2021 Jiang S et al. *JAMA Network Open.*

eTable 5. Propensity Modeling of Discharge Against Medical Advice Within 7 Days After Birth

Variable	Univariable Analysis			Multivariable Analysis*	
	OR (95%CI)	P-value		aOR (95%CI)	P-value
Gestational age	0.76 (0.73-0.78)	<0.001		0.83 (0.77-0.90)	<0.001
Birth weight	0.84 (0.83-0.86)	<0.001		0.90 (0.86-0.94)	<0.001
Male	0.72 (0.64-0.82)	<0.001		0.69 (0.59-0.81)	<0.001
SGA	1.33 (1.10-1.60)	0.003		1.37 (1.03-1.83)	0.031
Primigravida	0.91 (0.79-1.04)	0.151		0.96 (0.81-1.14)	0.643
Inborn	0.97 (0.85-1.11)	0.680		-	-
5-min Agpar score ≤3	3.04 (2.13-4.34)	<0.001		2.06 (1.33-3.20)	0.001
TRIPS score	1.04 (1.04-1.05)	<0.001		1.01 (1.00-1.02)	0.001
Cesrean section	0.49 (0.43-0.57)	<0.001		0.78 (0.65-0.94)	0.009
Prenatal care	0.38 (0.26-0.55)	<0.001		0.65 (0.37-1.15)	0.141
Antenatal steroids	0.43 (0.37-0.49)	<0.001		0.54 (0.45-0.64)	<0.001
Maternal hypertension	0.92 (0.76-1.11)	0.407		-	-
Maternal diabetes	0.54 (0.42-0.70)	<0.001		0.67 (0.50-0.91)	0.009

*Multilevel mixed-effects logistic regression model accounting for the correlations among the infants within sites

Abbreviations: OR, odds ratio; aOR adjusted odds ratio; 95%CI, 95% confidence interval; SGA, small for gestational age; TRIPS score, Transport Risk Index of Physiologic Stability score

© 2021 Jiang S et al. *JAMA Network Open.*

eTable 6. In-Hospital Outcomes and NICU Treatments of Matched Non-DAMA Infants for Infants DAMA in 7 Days

	Total	24 weeks	25 weeks	26 weeks	27 weeks	28 weeks	29 weeks	30 weeks	31 weeks	Total
	N=801	N=20	N=26	N=52	N=89	N=135	N=148	N=175	N=156	N=801
Survival without morbidity[a], n (%)	440 (54.9%)	1 (5.0%)	5 (19.2%)	23 (44.2%)	38 (42.7%)	60 (44.4%)	92 (62.2%)	116 (66.3%)	105 (67.3%)	440 (54.9%)
Survival rate, n (%)	636 (79.4%)	3 (15.0%)	12 (46.2%)	34 (65.4%)	59 (66.3%)	107 (79.3%)	122 (82.4%)	157 (89.7%)	142 (91.0%)	636 (79.4%)
IVH grade III or above or PVL[b], n/N (%)	86/701 (12.3%)	4/10 (40.0%)	3/19 (15.8%)	8/44 (18.2%)	14/74 (18.9%)	24/119 (20.2%)	11/135 (8.1%)	12/160 (7.5%)	10/140 (7.1%)	86/701 (12.3%)
NEC stage II or above, n (%)	50 (6.2%)	0 (0.0%)	2 (7.7%)	4 (7.7%)	4 (4.5%)	13 (9.6%)	10 (6.8%)	10 (5.7%)	7 (4.5%)	50 (6.2%)
BPD[c], n/N (%)	130/587 (22.1%)	2/3 (66.7%)	6/12 (50.0%)	7/31 (22.6%)	13/54 (24.1%)	25/101 (24.8%)	22/111 (19.8%)	28/142 (19.7%)	27/133 (20.3%)	130/587 (22.1%)
ROP stage III or above[d], n/N (%)	15/554 (2.7%)	2/4 (50.0%)	3/12 (25.0%)	2/33 (6.1%)	3/57 (5.3%)	2/95 (2.1%)	1/113 (0.9%)	2/135 (1.5%)	0/105 (0.0%)	15/554 (2.7%)

[a] Morbidities include NEC stage II or above, IVH grade III or above or PVL, BPD and ROP stage III or above

[b] Rate of IVH grade III or above or PVL was calculated among infants with neuroimaging results

[c] BPD was defined as mechanical ventilation or oxygen dependency at 36 weeks' postmenstrual age. Rate of BPD was calculated among infants with known respiratory support status at 36 weeks' postmenstrual age

[d] Rate of ROP was calculated among infants with eye examination in NICU

Abbreviations: DAMA, discharge against medical advice; 95%CI, 95% confidence interval; NEC, necrotizing enterocolitis; IVH, intraventricular hemorrhage; PVL, periventricular leukomalacia; BPD, bronchopulmonary dysplasia; ROP, retinopathy of prematurity

© 2021 Jiang S et al. *JAMA Network Open.*

eFigure 1. Rates of Discharge Against Medical Advice by Gestational Age

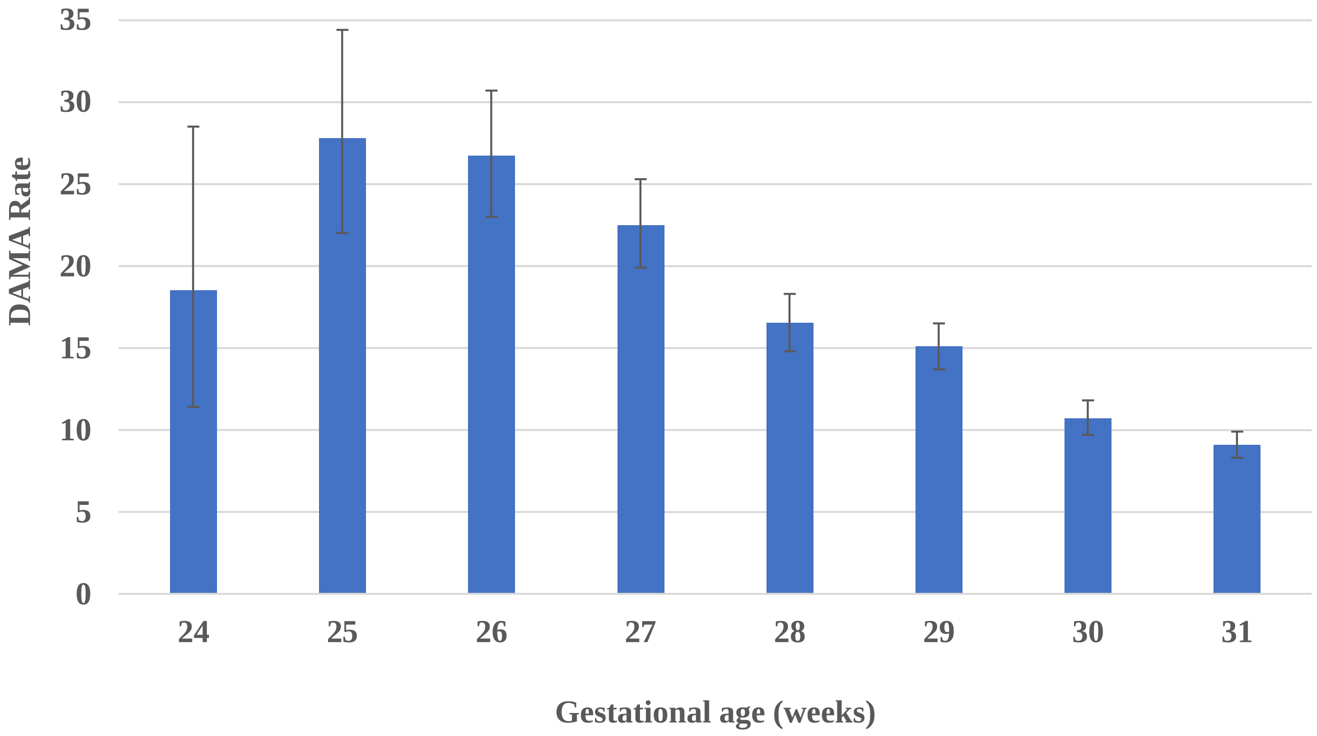

© 2021 Jiang S et al. *JAMA Network Open.*

eFigure 2. Incidences of Neonatal Outcomes With 95% Confidence Intervals Among Matched Non-DAMA Infants and all Non-DAMA Infants

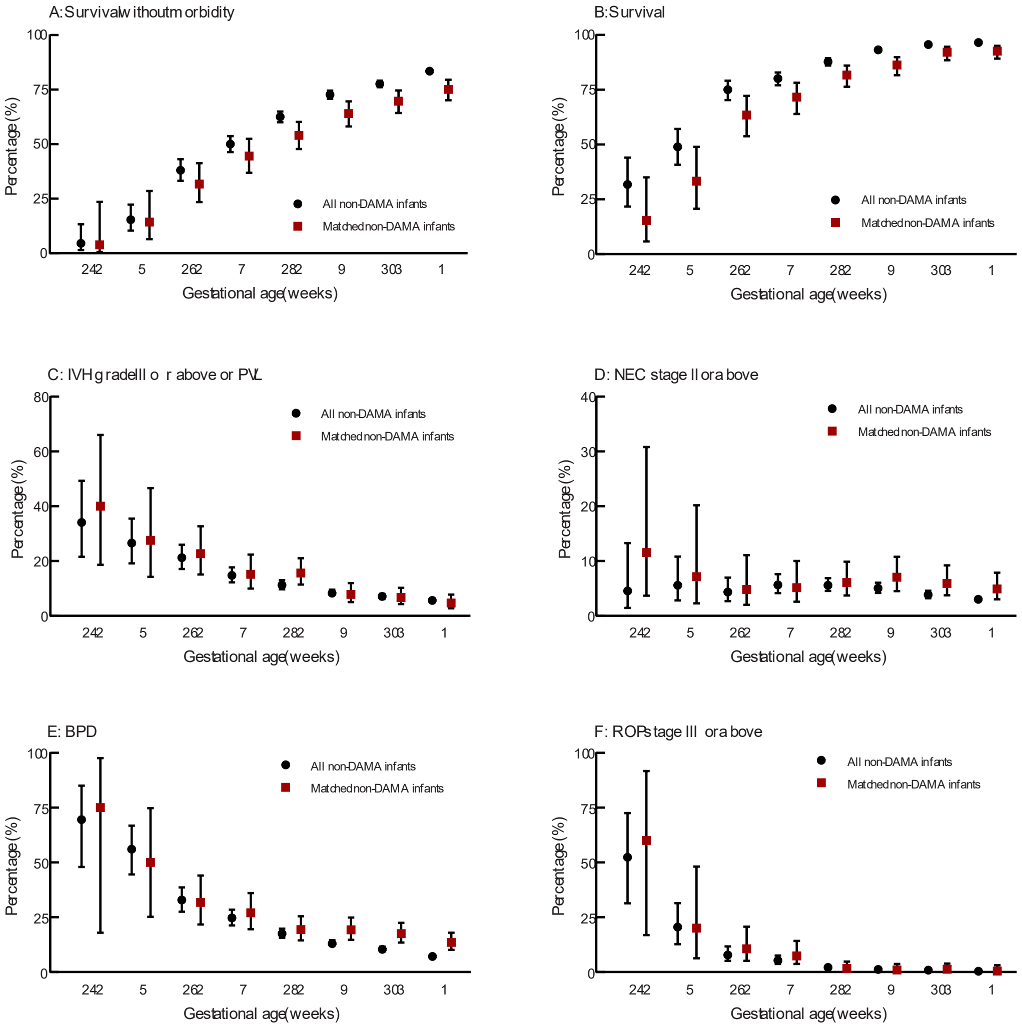

© 2021 Jiang S et al. *JAMA Network Open.*

© 2021 Jiang S et al. *JAMA Network Open.*
