# Supplementary material for: Estimated Survival and Major Comorbidities of Very Preterm Infants Discharged Against Medical Advice vs Treated With Intensive Care in China
Source: JAMA Netw Open. 2021 Jun 17;4(6):e2113197. doi: 10.1001/jamanetworkopen.2021.13197 (PMC12543406; doi:10.1001/jamanetworkopen.2021.13197)
Supplement: Supplement 2. — Reduction of Infection in Neonatal Intensive Care Units Using the Evidence-Based Practice for Improving Quality (REIN-EPIQ) Study Group Members and Site Investigators [file jamanetwopen-e2113197-s002.pdf]

Supplemental Online Content: Nonauthor Collaborators

*Indicates required information. Only first name, last name, and suffix will appear in PubMed.

*Group Name(s): Reduction of Infection in Neonatal Intensive Care Units Using the Evidence-Based Practice for Improving Quality (REIN-EPIQ) Study Group							
***First Name and Middle Initial(s)**	***Last Name**	***Suffix (eg, Jr, III)**	Academic Degrees	Institution	Location (city, state/province, country)	Role or Contribution, eg, chair, principal investigator	Group (if more than 1 Group listed in the byline) and/or Subgroup (eg, Steering Committee)
**REIN-EPIQ Study Group**							
Yun	Cao		MD, PhD	Children's Hospital of Fudan University	Shanghai, China	Group Member	REIN-EPIQ Study Group
Siyuan	Jiang		MD, PhD	Children's Hospital of Fudan University	Shanghai, China	Group Member	REIN-EPIQ Study Group
Yong	Ji		MD	Children's Hospital of ShanXi/Women's Health Center of Shanzi	Shanxi, China	Group Member	REIN-EPIQ Study Group
Shuping	Han		MD	Women's Hospital of Nanjing Medical University	Jiangsu, China	Group Member	REIN-EPIQ Study Group
Sannan	Wang		MD	Suzhou Municipal Hospital	Jiangsu, China	Group Member	REIN-EPIQ Study Group
Zhankui	Li		MD	Northwest Women and Children's Hospital	Shaanxi, China	Group Member	REIN-EPIQ Study Group
Shiwen	Xia		MD	Women and Children’s Hospital of Hubei Province	Hubei, China	Group Member	REIN-EPIQ Study Group
Changyi	Yang		MD	Fujian Provincial Maternity and Children's Hospital/Affiliated Hospital of Fujian Medical University	Fujian, China	Group Member	REIN-EPIQ Study Group
Chuanzhong	Yang		MD	The Affiliated Shenzhen Maternity and Child Healthcare Hospital of Southern Medical University	Guangdong, China	Group Member	REIN-EPIQ Study Group
Ling	Chen		MD	Tongji Hospital, Tongji Medical College, Huazhong University of Science and Technology	Hubei, China	Group Member	REIN-EPIQ Study Group
Ruobing	Shan		MD	Qingdao Women and Children’s Hospital	Shandong, China	Group Member	REIN-EPIQ Study Group
Ling	Liu		MD	Guiyang Maternal and Child Health Care Hospital	Guizhou, China	Group Member	REIN-EPIQ Study Group

Supplemental Online Content: Nonauthor Collaborators

*Indicates required information. Only first name, last name, and suffix will appear in PubMed.

*First Name and Middle Initial(s)	*Last Name	*Suffix (eg, Jr, III)	Academic Degrees	Institution	Location (city, state/province, country)	Role or Contribution, eg, chair, principal investigator	Group (if more than 1 Group listed in the byline) and/or Subgroup (eg, Steering Committee)
Bin	Yi		MD	Gansu Provincial Maternity and Child-care Hospital	Giansu, China	Group Member	REIN-EPIQ Study Group
Yang	Wang		MD	The First Affiliated Hospital of Anhui Medical University	Anhui, China	Group Member	REIN-EPIQ Study Group
Jiangqin	Liu		MD	Shanghai First Maternity and Infant Hospital, Tongji University School of Medicine	Hubei, China	Group Member	REIN-EPIQ Study Group
Ling	He		MD	Jiangxi Provincial Children’s Hospital	Jiangxi, China	Group Member	REIN-EPIQ Study Group
Mingxia	Li		MD	First Affiliated Hospital of Xinjiang	Xianjiang, China	Group Member	REIN-EPIQ Study Group
Xinnian	Pan		MD	The Maternal and Child Health	Guangxi. China	Group Member	REIN-EPIQ Study Group
Yan	Guo		MD	Children’s Hospital of Nanjing	Jiangsu, China	Group Member	REIN-EPIQ Study Group
Cuiqing	Liu		MD	Children’s Hospital of Hebei	Heibi, China	Group Member	REIN-EPIQ Study Group
Qin	Zhou		MD	The Affiliated Wuxi Maternity and	Jiangsu, China	Group Member	REIN-EPIQ Study Group
Xiaoying	Li		MD	Qilu Children’s Hospital of	Shandong, China	Group Member	REIN-EPIQ Study Group
Hong	Xiong		MD	Children’s Hospital Affiliated to	Henan, China	Group Member	REIN-EPIQ Study Group
Yujie	Qi		MD	Children’s Hospital Affiliated to	Beijing, China	Group Member	REIN-EPIQ Study Group
Mingyan	Hei		MD	The Third Xiangya Hospital of	Hunan, China	Group Member	REIN-EPIQ Study Group
**REIN-EPIQ Study Group Site Investigators**							
Yong	Ji			Children’s Hospital of ShanXi / Women’s Health Center of Shanxi	Shanxi, China	Site Investigator	REIN-EPIQ Study Group
Yong	Han			Women’s Hospital of Nanjing Medical University	Jiangsu, China	Site Investigator	REIN-EPIQ Study Group
Shuping	Wang			Suzhou Municipal Hospital	Jiangsu, China	Site Investigator	REIN-EPIQ Study Group
Sannan	Li			Northwest Women and Children’s Hospital	Shaanxi, China	Site Investigator	REIN-EPIQ Study Group
Zhankui	Xia			Women and Children's Hospital of Hubei Province	Hubei, China	Site Investigator	REIN-EPIQ Study Group

Supplemental Online Content: Nonauthor Collaborators

*Indicates required information. Only first name, last name, and suffix will appear in PubMed.

***First Name and Middle Initial(s)**	***Last Name**	***Suffix (eg, Jr, III)**	Academic Degrees	Institution	Location (city, state/province, country)	Role or Contribution, eg, chair, principal investigator	Group (if more than 1 Group listed in the byline) and/or Subgroup (eg, Steering Committee)
Shiwen	Yang			Fujian Provincial Maternity and Children's Hospital/Affiliated Hospital of Fujian Medical University	Fujian, China	Site Investigator	REIN-EPIQ Study Group
Changyi	Yang			The Affiliated Shenzhen Maternity and Child Healthcare Hospital of Southern Medical University	Guangdong, China	Site Investigator	REIN-EPIQ Study Group
Chuanzhong	Lin			The Second Affiliated Hospital & Yuying Children's Hospital, Wenzhou Medical University	Wenzhou, China	Site Investigator	REIN-EPIQ Study Group
Zhenlang	Chen			Tongji Hospital, Tongji Medical College, Huazhong University of Science and Technology	Hubei, China	Site Investigator	REIN-EPIQ Study Group
Ling	Shan			Qingdao Women and Children’s Hospital	Shandong, China	Site Investigator	REIN-EPIQ Study Group
Ruobing	Liu			Guiyang Maternal and Child Health Care Hospital	Guizhou, China	Site Investigator	REIN-EPIQ Study Group
Ling	Yi			Gansu Provincial Maternity and Child-care Hospital	Gansu, China	Site Investigator	REIN-EPIQ Study Group
Bin	Wang			The First Affiliated Hospital of Anhui Medical University	Anhui, China	Site Investigator	REIN-EPIQ Study Group
Yang	Liu			Shanghai First Maternity and Infant Hospital, Tongji University School of Medicine	Shanghai, China	Site Investigator	REIN-EPIQ Study Group
Jiangqin	He			Jiangxi Provincial Children's Hospital	Jiangxi, China	Site Investigator	REIN-EPIQ Study Group
Ling	Li			First Affiliated Hospital of Xinjiang Medical University	Xinjiang, China	Site Investigator	REIN-EPIQ Study Group
Mingxia	Pan			The Maternal and Child Health Hospital of Guangxi Zhuang Autonomous Region	Guangxi, China	Site Investigator	REIN-EPIQ Study Group

*Indicates required information. Only first name, last name, and suffix will appear in PubMed.

***First Name and Middle Initial(s)**	***Last Name**	***Suffix (eg, Jr, III)**	Academic Degrees	Institution	Location (city, state/province, country)	Role or Contribution, eg, chair, principal investigator	Group (if more than 1 Group listed in the byline) and/or Subgroup (eg, Steering Committee)
Xinnian	Guo			Children’s Hospital of Nanjing Medical University	Jiangsu, China	Site Investigator	REIN-EPIQ Study Group
Yan	Liu			Children’s Hospital of Hebei Province,	Hebei, China	Site Investigator	REIN-EPIQ Study Group
Cuiqing	Zhou			The Affiliated Wuxi Maternity and Child Health Hospital of Nanjing Medical University	Jiangsu, China	Site Investigator	REIN-EPIQ Study Group
Qin	Li			Qilu Children’s Hospital of Shandong University	Shandong, China	Site Investigator	REIN-EPIQ Study Group
Xiaoying	Xiong			Children’s Hospital Affiliated to Zhengzhou University	Henan, China	Site Investigator	REIN-EPIQ Study Group
Hong	Qi			Beijing Children’s Hospital of Capital Medical University	Beijing, China	Site Investigator	REIN-EPIQ Study Group
Yujie	Hei			The Third Xiangya Hospital of Central South University	Hunan, China	Site Investigator	REIN-EPIQ Study Group
